# Mitochondrial DNA Replacement Techniques to Prevent Human Mitochondrial Diseases

**DOI:** 10.3390/ijms22020551

**Published:** 2021-01-07

**Authors:** Luis Sendra, Alfredo García-Mares, María José Herrero, Salvador F. Aliño

**Affiliations:** 1 Unidad de Farmacogenética, Instituto de Investigación Sanitaria La Fe, 46026 Valencia, Spain; luis.sendra@uv.es (L.S.); alino@uv.es (S.F.A.); 2 Departamento de Farmacología, Facultad de Medicina, Universidad de Valencia, 46010 Valencia, Spain; algarma2@alumni.uv.es; 3 Unidad de Farmacología Clínica, Área del Medicamento, Hospital Universitario y Politécnico La Fe, 46026 Valencia, Spain

**Keywords:** mitochondrial diseases, mitochondrial DNA, mitochondrial replacement, mitochondrial donation, three-parent baby

## Abstract

**Background:** Mitochondrial DNA (mtDNA) diseases are a group of maternally inherited genetic disorders caused by a lack of energy production. Currently, mtDNA diseases have a poor prognosis and no known cure. The chance to have unaffected offspring with a genetic link is important for the affected families, and mitochondrial replacement techniques (MRTs) allow them to do so. MRTs consist of transferring the nuclear DNA from an oocyte with pathogenic mtDNA to an enucleated donor oocyte without pathogenic mtDNA. This paper aims to determine the efficacy, associated risks, and main ethical and legal issues related to MRTs. **Methods:** A bibliographic review was performed on the MEDLINE and Web of Science databases, along with searches for related clinical trials and news. **Results:** A total of 48 publications were included for review. Five MRT procedures were identified and their efficacy was compared. Three main risks associated with MRTs were discussed, and the ethical views and legal position of MRTs were reviewed. **Conclusions:** MRTs are an effective approach to minimizing the risk of transmitting mtDNA diseases, but they do not remove it entirely. Global legal regulation of MRTs is required.

## 1. Introduction

Mitochondria are double membrane organelles present in most eukaryotic cells [1], whose main function is to obtain energy as ATP (Adenosine 5’-triphosphate) through oxidative phosphorylation (OXPHOS) [2]. The OXPHOS process involves five multi-enzymatic complexes that couple the electron transport chain with the synthesis of ATP [1]. Besides providing up to 95% of the energy requirements of cells [3,4,5], OXPHOS also participates in other processes, such as thermogenesis, calcium homeostasis, programmed cell death (apoptosis), and the secondary production of reactive oxygen species (ROS) [1,6]. Mitochondria are semi-autonomous and contain their own genome: the mitochondrial DNA or mtDNA [7]. The mtDNA molecule is double-stranded, circular, and contains approximately 16.6 kb [2,8]. Each mitochondria carries 2 to 10 copies of mtDNA [1]; this implies that a human oocyte can contain from 100, to more than 100,000, copies of mtDNA [9]. Despite their multi-copy nature, the mtDNA only represents 0.1% of a cell genome [10]. The mtDNA is inherited exclusively from the mother since at fertilization, the mitochondria of the sperm are degraded and the ovum provides all the zygote mitochondria.

Mitochondrial genetics is very complex and requires the coordinated expression of genes located in both mtDNA and nDNA [5,7,11]. In fact, more than 1000 mitochondrial proteins are encoded by nDNA [5]. Human mtDNA contains 37 genes: 22 genes encode transfer RNA, 2 encode ribosomal RNA, and the other 13 genes encode polypeptides involved in OXPHOS [2,10,12]. The rest of the necessary proteins for the correct function of mitochondrion, including 79 structural subunits of the OXPHOS [13], are encoded by genes within nuclear DNA (nDNA), and are imported from the cytoplasm [6,14]. Characteristically, mtDNA presents a high mutation rate [11], and these mutations constitute the most frequent inherited metabolic diseases [6]. Mitochondrial diseases are a group of genetic pathologies characterized by presenting dysfunctional mitochondria with defective OXPHOS process [6,15] and ATP production, which mainly affect tissues with higher energy consumption [4,5]. This review focuses only on those mitochondrial diseases caused by pathogenic mutations within the mtDNA. One in 200 live births carries a mutated mtDNA and, despite all not developing the disease, women will transmit the mutation to their offspring [16]. The prevalence of mitochondrial diseases due to mtDNA mutations is approximately 1/5000 [17]. Mutated and non-mutated mtDNA molecules coexist in the cells of an individual, a phenomenon called heteroplasmy [5,7]. The higher the percentage of mutated mtDNA molecules, the higher is the level of heteroplasmy (from 0% to 100%) [7]. It is assumed that a mitochondrial disease clinically appears when heteroplasmy achieves levels over 60%. From this threshold, the disease severity increases as heteroplasmy does [6]. Threshold theory does not define the value of the heteroplasmy, but claims the presence of a biochemical threshold line, which depends upon individual cells [5]. Women carrying mutated mtDNA have different options to have free-of-disease offspring: (a) adoption; (b) gestation with a non-related donor ovum; (c) pre-implantation genetic diagnostic (blastocysts biopsy) to select healthy embryos; and (d) pre-natal genetic diagnostic (amniotic biopsy) with the option of pregnancy interruption.

Only options (c) and (d) would permit mutation carriers to have genetically related healthy offspring. In women carrying deleterious mtDNA mutations wanting to have offspring, ovum and embryos with low heteroplasmy levels must be selected. However, the risk of offspring developing mitochondrial pathology does not disappear, due to bottleneck and random genetic drift. The bottleneck theory suggests that, during the female embryo development, the precursor cells of oocytes reduce their amount of mtDNA molecules. Later, daughter cells amplify selectively the scarce molecules of mtDNA until obtaining the high amounts of mtDNA present in mature oocytes, causing heteroplasmy disparity among the oocytes of the same woman [5,16], enabling the transmission of a mitochondrial disease to offspring. In “random genetic drift”, heteroplasmy levels changes over time, especially during embryo development due to relaxation in the mitochondrial cycle (asymmetric distribution of mitochondria to daughter cells during mitosis, relaxed mtDNA replication) [4,7,18]. As a consequence, when an embryo is generated from an heteroplasmic oocyte, the final level of mutated DNA can differ greatly among the tissues of the same individual, supporting the phenotypical variety observed in mitochondrial diseases (including the absence of symptoms) [4,7].

Different mitochondrial replacement techniques (MRTs) have been proposed with the aim of completely preventing the vertical transmission of mtDNA pathologies [15], and substituting the entire mitochondrial repertoire (with a mutated genome) of an oocyte/zygote for another functional one. Generally, MRTs consist in transferring the nDNA from an oocyte or zygote containing mutated mtDNA to an oocyte or zygote with functional mitochondria, previously enucleated. The resulting zygote has nDNA from the biological mother and cytoplasmic material, including healthy mitochondrion, from the donor oocyte [19,20,21]. At present, it is estimated that MRTs could prevent 150 cases of mitochondrial diseases in the UK and, approximately, 780 in the US [22]. Besides the evident benefits of these techniques to the families affected by the disease, they could also greatly affect health expenditure. However, there is not much information about their safety. The only precedent available in the literature about mitochondrial donations is focused on the ooplasmic transfer performed in the USA between 1997 and 2001 [23]. The goal of this strategy was that the cytoplasmic factors transferred, including mitochondrion, could improve the fertility of the recipient oocyte [24]. After more than 30 births, the FDA banned this technique in 2001 due to the occurrence of congenital defects and potential ethical issues derived from the germline modification [24,25]. In this sense, MRTs are not exempt from ethical objections (use of embryos for research purposes, germline modifications, etc.). In 2015, the UK was a pioneer in approving the clinical use of MRTs for the first time. The application of these strategies has extended to other countries, although without the appropriate legislation [26]. This review aims to evaluate the safety, efficacy, and ethics of MRTs jointly, a need that has not been covered to date in the literature.

## 2. Results

### 2.1. Mitochondrial Replacement Procedures and Variables Measured

In the reviewed literature, five techniques for mitochondrial replacement therapies with clinical trial support were found:

1. Germinal Vesicle Transfer (GVT) [27,28]
2. Maternal Spindle Transfer (MST) [19,27,28,29,30,31,32,33,34]
3. Pronuclear Transfer (PNT) [20,27,28,31,35,36,37]
4. Polar Body Transfer:
  - First Polar Body Transfer (PB1T) [31,38,39,40]
  - Second Polar Body Transfer (PB2T) [31,38,40]

**Figure 1:**
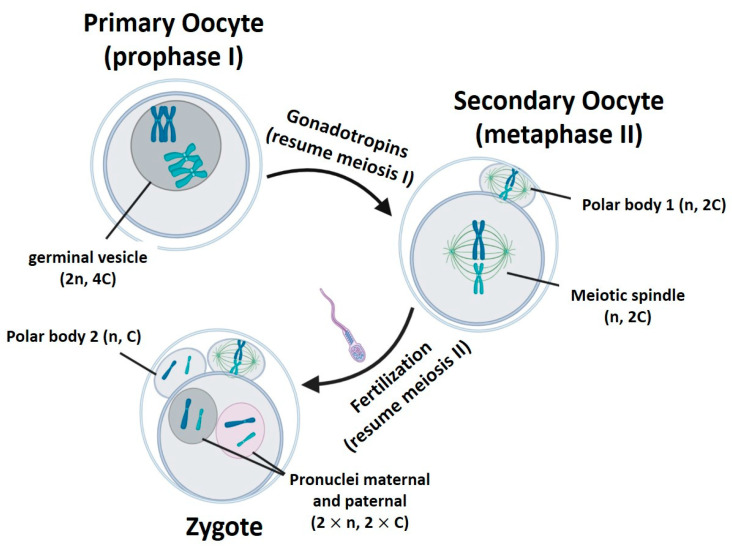
Gametogenesis process. n represents the number of chromosome sets. C is the amount of chromatids in a chromosome pair. Created with BioRender.com.

The main difference among the techniques is the biological form of the nuclear genome when it is transferred within the karyoplast, considering the changes occurring in oocyte nDNA during the gametogenesis (Table 1, Figure 1).

Two main variables have been employed by researchers to evaluate the safety and efficacy of MRTs:

(1) Potential embryo development of the reconstituted oocyte/zygote: this variable informs about the survival rate of the reconstituted oocyte/zygote after its micromanipulation, and its potential to progress to different development stages (morula, blastocyst, or even birth in animals) [19,27,28]. In humans, generally the development reached morula or blastocyst stages and fetuses survived only to mid-gestation [1,35]. However, another work [34] reported the birth of a healthy boy from a mother carrying mutated mtDNA. Given the ethical and legal restrictions on human embryo research, many authors decided to deeply study the potential development of embryonic stem cells (ESCs) from blastocysts [29,30,32,33,36,37,39,40], although these cells may not be representative of normal embryo cells [1].
(2) Mitochondrial DNA carry-over: mitochondrial carry-over is defined as the mtDNA that is inadvertently transferred with the nDNA, since, to date, 100% mitochondrial exclusion has not been possible. This carry-over is expressed as heteroplasmy percentage: the ratio of maternal mtDNA with respect to the donor ovum mtDNA.

Mitochondrial DNA carry-over is undesirable as it supposes that mutant mtDNA has been transferred along with nDNA, allowing the risk that offspring could develop the mitochondrial disease. In this respect, rates of carry-over higher than 5% have proved to increase the risk of developing mitochondrial diseases in posterior generations (in females), and carry-over rates over 20% increased the risk of presenting a mitochondrial disease throughout life [36]. For this reason, the goal in every research work has been obtaining the lowest possible rates of carry-over, even under levels of 2–3% [19,20], in order to avoid potential deleterious effects. Embryonic stem cell derivation plays an important role in carry-over studies, since cell sub-cultures permit evaluating the evolution of the carried-over mtDNA over time, and somehow emulating the normal embryo development [32,33,36].

### 2.2. Germinal Vesicle Transfer (GVT)

**Figure 2:**
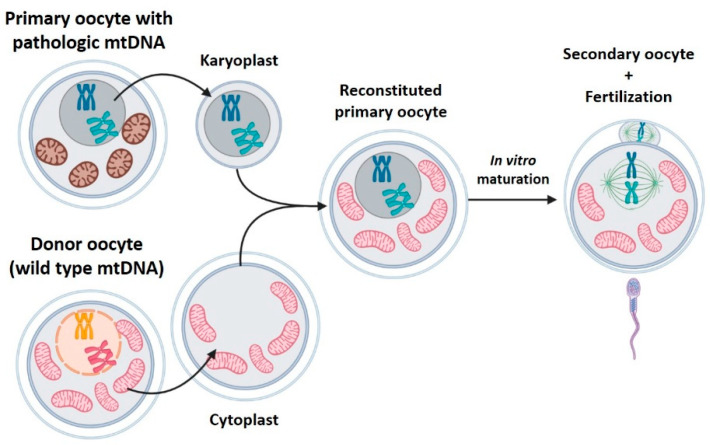
Germinal vesicle transfer (GVT). GVT consists of transplanting the germinal vesicle from a patient’s primary oocyte to a donor oocyte without its germinal vesicle. Created with BioRender.com.

The germinal vesicle is the large nucleus present in immature (primary) oocytes. Primary oocytes remain arrested in prophase 1, with the nucleus as germinal vesicle within the ovary, for years until they are stimulated in successive menstrual cycles, after puberty. GVT consists in transplanting the germinal vesicle from a patient’s primary oocyte to a donor oocyte in which the germinal vesicle has also been extracted (Figure 2). Since these are immature oocytes, the resulting transplanted oocyte will be subjected to a maturation process in vitro to complete the meiosis. After that, it will be fertilized and implanted [27,28].

Only two works have evaluated GVT [27,28], both in murine models. Their main results are shown in Table 2. The low efficacy of this technique in generating offspring is striking. In fact, in the work of Neupane et al., 2014 [28] no embryo survived until the blastocyst stage. This is because GVT uses primary immature oocytes that, after being reconstructed, must undergo an in vitro maturation process to continue the meiosis before they are fertilized. The techniques for in vitro maturation available today, reduce the potential development of oocytes [19]. Given the poor results obtained in animal models, it is assumed that this technique will not be employed in human oocytes until the in vitro maturation procedures are optimized.

### 2.3. Meiotic Spindle Transfer (MST)

**Figure 3:**
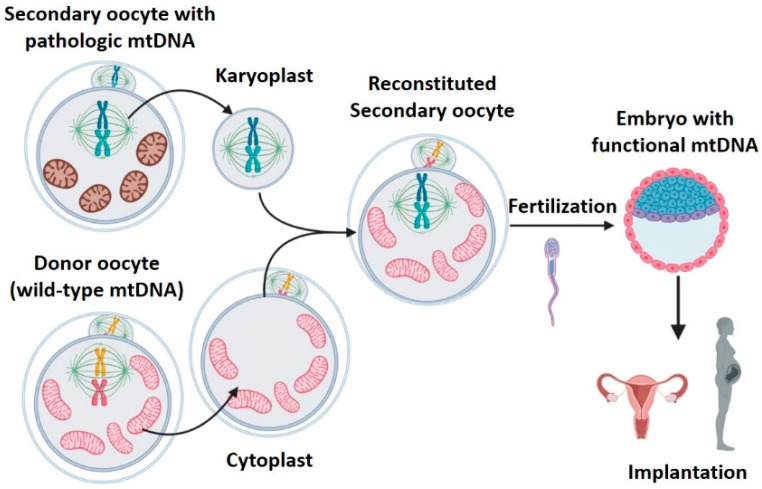
Meiotic spindle transfer (MST). MST consists of transferring the meiotic spindle of a patient’s metaphase II oocyte (with mitochondrial disease) to a healthy enucleated donor oocyte. Created with BioRender.com.

During ovulation, the increase of gonadotropins provokes the primary oocyte to resume and complete the meiotic division, resulting in the secondary oocyte, which starts the second meiotic division. This process stops at metaphase II, until it is fertilized [40]. During metaphase II, the oocyte nucleus is not defined and chromosomes are located within the meiotic spindle. The lack of nuclear envelope makes it difficult to observe the nDNA in this phase. However, it is possible to observe the meiotic spindle with a polarized light microscope, thanks to the birefringent properties of microtubules [19]. MST consists in transferring the meiotic spindle of a patient’s oocyte in metaphase II (with mitochondrial disease) to a healthy donor oocyte, previously enucleated (Figure 3). Then, the reconstituted oocyte will be fertilized and implanted [19,27,29,30,31,32,34,41].

**Figure 4:**
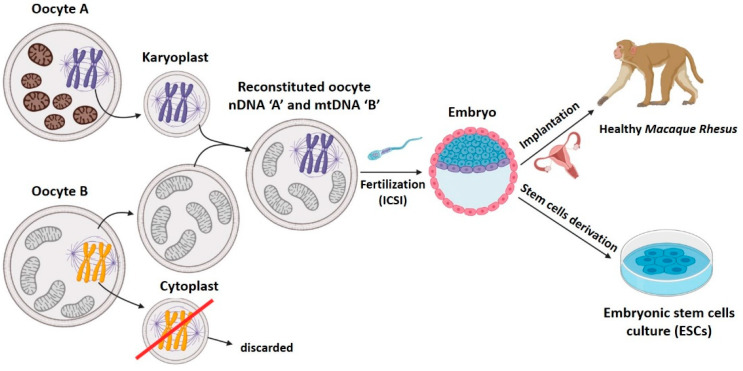
The meiotic spindle transfer technique described by Tachibana et al. [19] in 2009 that would be employed, with modifications, in many later works. Created with BioRender.com.

Meiotic spindle transfer was described recently, but it has already accumulated numerous articles employing both animal [19,27,28,31] and human [29,30,32,33,34] gametes. Among the works employing human gametes, one describes the birth of a healthy male descendant of a mother carrying pathologic mtDNA [34]. The article published by Tachibana et al. [19] in 2009 was a milestone, since it described in detail the MST technique that would be employed, with modifications, in many later works (Figure 4). In this paper, the authors transferred the metaphase II meiotic spindle between two oocytes from rhesus macaques. To achieve the fusion of karyoplast and cytoplast, they employed HVJ-E proteins. Later, the reconstituted oocytes were fertilized by intracytoplasmic sperm injection (ICSI), and transplanted to the uterus of females of the same species, resulting in four completely healthy baby primate births [19,30]. Tachibana et al. demonstrated that MST was a viable MRT since it was safe and effective, and was proved by:

1. High efficacy: reconstituted oocytes presented rates of development to blastocyst equivalent to those obtained in controls [19]. Furthermore, the four macaques born remained healthy in both physical (weight, height, etc.) and analytical explorations during 3 years follow-up [30]. MST was compatible with normal embryo growth, and was able to generate healthy individuals.
2. Low mitochondrial carry-over from biological mother: it was lower than 3% [19] in tissue samples from all rhesus macaques born in the study, and these levels remained stable, and always at 2%, over a 19-month period [30].

Generally, the rest of the papers employing MST in animal models also achieved embryo development rates similar to those observed in non-manipulated controls [27,28,31], and low levels of mitochondrial carry-over [28] (Table 3). As an exception, in the work of Wang et al. in 2014 [31], the carry-over was higher, with average levels of 5.5% and 7.7% in tissue samples from first and second generation mice, respectively. This was probably due to the participation of less-skilled microinjection operators, since the paper aimed to highlight the advantages of another mitochondrial replacement technique. It is important to remark that only one other study (Tachibana et al., 2009) evaluated the tissues of animals obtained with this strategy, and only in the first offspring generation. Thus, further preclinical studies and results will be required for determining the behavior of long-term mitochondrial carry-over in successive generations.

The promising results achieved with MST by Tachibana in 2009 in rhesus macaques [19] supported the translation to a human setting by the same research group, employing oocytes from healthy donors [30]. Researchers transferred the meiotic spindle between human oocytes (*n* = 64), which were fertilized later by ICSI and cultured in vitro until blastocyst stage (Table 4). Besides the blastocysts obtained by MST, five embryonic stem cell (ESCs) lines were derived to evaluate the mitochondrial carry-over, and detect potential chromosomal anomalies. As already explained, the ESCs derivation permits the researchers working with human gametes to simulate their further embryo development and avoid the ethical–legal concerns existing in research with human embryos over 14 days (“14-day rule”) [9]. Despite the normal ESCs phenotype and good carry-over rates obtained (below 1% in oocytes and ESCs, Table 4) [30], the authors detected a lower level of development to blastocyst compared with that observed in macaques (43% vs. 61%) [19,30]. The reason was that over 50% of the human zygotes obtained presented abnormal fertilization, an anomalous number of pronuclei within the zygote, that did not occur in macaques [30]. The abnormal fertilization increases the risk of aneuploidy in embryo and worsens its potential development, and diminishing the procedure efficacy. Researchers tried to elucidate the cause of the abnormal fertilization by modifying the technique, but this was not possible. This is a clear example that, although pre-clinical studies in animals provide very valuable information, the results obtained are not always applicable in humans, not even when they are performed in species evolutionarily close to humans, such as primates. Later, three more articles proved the viability of MST in human oocytes (Table 4) [29,32,33]. It again must be highlighted, that a low number of oocytes reached the blastocyst stage in the studies of Paull et al., 2013 [29] and Yamada et al. 2016 [32], both published by the same group. Rates were even lower than those obtained by Tachibana et al., already discussed. Instead of fertilizing the oocytes by ICSI, the authors fertilized them in a parthenogenetic manner (parthenotes zygotes) I/E, without the participation of spermatozoids [29,32]. Parthenogenesis is an artificial way of activating oocytes that avoids creating embryos for research purposes, although their development capacity is reduced [29]. The development of parthenotes to blastocyst was similar to that achieved by controls [29,32], suggesting that the MST technique designed by Tachibana [19,30] does not affect the embryo development, and can be successfully replicated in independent laboratories.

On the other hand, these three studies carried out on humans obtained very low rates of mitochondrial carry-over in the generated embryos (<1%, Table 4) [29,32,33]. However, when evaluating the carry-over in prolonged cultures of ESCs derived from blastocysts, mitochondrial genotype instability was reported. Yamada et al. 2016 [32] derived eight ESC lines from blastocysts obtained with MST. Seven of these lines had undetectable levels of carry-over during 6-month culture (over 30 passages). In contrast, in the other line, the carried mtDNA showed an increase from 1% (passage 1) to a maximum of 53% (passage 36). Then, the carry-over level decreased until 1% in passage 59 [32]. Kang et al. 2016 [33] derived up to 18 ESCs lines, three of them obtained from embryos with spindle transfer in oocytes carrying mtDNA pathogenic mutations. After a short period of culture, three out of these 18 ESCs lines completely reverted to the mitochondrial haplotype carried over I/E, and 100% of their mtDNA happened to be that of their biological mother. Curiously, despite one of these reverted lines coming from an oocyte with mutated mtDNA, the mutation did not appear [33]. It has been suggested that some mitochondrial haplotypes could have some replicative advantages that could explain the mtDNA reported in different articles [33]. Furthermore, ESCs might not show the same mtDNA behavior as normal embryos and their utility as a developmental model could be limited [34,36]. However, these findings are worrying: even trace amounts of mitochondrial carry-over (less than 1%) [32,33] could amplify in tissues during embryo development or postnatal growth. Whether or not this occurred with mutated mtDNA, the individual would be at risk of presenting mitochondrial pathology.

A recent work about MST in humans reported the birth of an apparently healthy male, the son of a woman carrying a deleterious mutation in mtDNA (m.8993T > G) responsible for Leigh syndrome [34]. This constitutes the first human birth derived from the clinical application of a mitochondrial replacement technique. In this study, the high efficacy observed in blastocysts generation is surprising (80% of all fertilized oocytes), much higher than that observed in other works performed on humans [19,29,30,32,33]. In any case, the obtained blastocysts had a low quality, and only one was free of aneuploidies, which was transferred to the maternal uterus and successfully implanted [34]. Gestation and childbirth proceeded normally and the neonate remained healthy during the 7-month follow-up. The carry-over rate varied widely among the samples from neonatal tissues, maybe due to an unknown mechanism of selective amplification (random genetic drift) [34]. Despite the rate of carry-over being substantially higher than observed in other works of MST in humans [19,29,30,32,33], the levels were much lower than the threshold (>60%) from which the disease phenotype appears [30], and therefore, it is probable that the child will not develop the disease [34]. The authors proposed a long-term follow-up of mutated mtDNA presence in tissues, but the family rejected future controls.

In summary, MST is compatible with embryonic development and has generated healthy births in both animals (primates and mice), and one healthy birth in humans. Despite the low rates of carry-over derived from the technique, there is a risk that the carried mtDNA will increase in the offspring. There is a lack of studies, particularly in animals, that permit determining the long-term implications of this technique.

### 2.4. Pronuclear Transfer (PNT)

**Figure 5:**
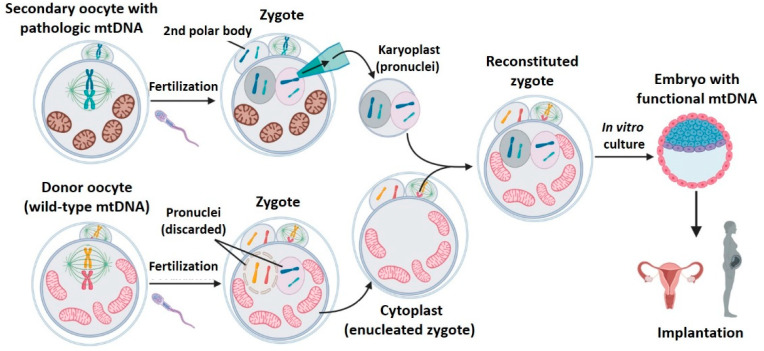
Pronuclear transfer (PNT). During PNT, the pronuclei of a patient’s oocyte is isolated in a karyoplast and transferred into a donor oocyte without its pronucleus. Created with BioRender.com.

After being fertilized by a spermatozoid, the metaphase II oocyte resumes its maturation process and finishes the second meiotic division. This zygote presents its genetic material organized into two united but clearly differentiated pronuclei, one of maternal origin, and the other paternal. During PNT, the pronuclei of a patient’s oocyte is isolated in a karyoplast and is transferred into a donor oocyte, whose pronuclei has been previously removed [20,25,28,31,32,35,37,42,43] (Figure 5). Contrary to GVT and MST techniques, this strategy requires the creation and manipulation of zygotes.

Briefly, PNT consists in transferring the pronuclei from a zygote to another enucleated one. For this reason, the main inconvenience of this technique is ethical: the nuclear transfer requires generating two embryos, and discarding one of them. Table 5 (animals) and Table 6 (humans) compile the PNT results found. PNT is the mitochondrial replacement technique with the longest tradition in the scientific literature: the first work found dates from 1983 [42]. Authors demonstrated in mice that PNT was compatible with embryonic development and obtained 10 animals with normal phenotype [42]. Later, many other studies in murine model confirmed the efficacy of PNT to generate embryos [27,28,31,43]. Sato et al. 2005 [43] prevented the vertical transmission of mitochondrial disease in a murine model with pathogenic mtDNA deletions. Despite the mice obtained being free of disease, mitochondrial carry-over (Table 5) was quite high (11%), and it increased until 12%, on average, at day 300 [43]. The mitochondrial carry-over observed in the rest of the works was very inconsistent, from minimum, even undetectable, levels in 84% of tested embryos in Neupane et al. 2014 [28], to rates close to 24% in Wang et al. 2014 [31]. PNT has proved to be able to achieve low levels of carry-over, but the great variability observed suggests that this technique is too dependent on the operator and the micromanipulation procedure employed.

Pronuclear transfer has also been successfully performed on human zygotes (Table 6) [20,35,36,37], although it did not lead to any live births. The first work that studied the technique in humans [20], employed abnormally fertilized zygotes discarded from cycles of in vitro fertilization. The use of zygotes whose fertilization had been anomalous could explain the scarce evolution to blastocyst found (8.3%) [20], although it is still a low rate when compared to that obtained in another study that employed abnormally fertilized zygotes, but with spindle transfer [30]. Later, the same research group could significantly improve the embryonic development potential of PNT by introducing different technical modifications [36], such as changing the composition of the manipulation medium and reducing the concentration of inactivated Sendai virus proteins employed for the fusion.

The levels of mitochondrial carry-over observed in works of PNT on human embryos were low in both blastocysts and embryonic stem cells (Table 6) [20,35,36,37]. Among them, Graven et al., 2010 [20] and Hyslop et al., 2016 [36] reported lower levels of carry-over, due to performing an extremely careful micromanipulation to reduce, as much as possible, the amount of cytoplasm transferred with the biological mother nDNA within the karyoplast. These findings are in agreement with those already discussed from animal models of PNT [28,31,43]: mitochondrial carry-over during pronuclear transfer greatly depends on the operator.

Surprisingly, Hyslop et al. 2016 [36] detected that mitochondrial genotype was unstable in prolonged cultures of ESCs derived from embryos subjected to PNT. One of these ESCs line suffered a progressive increase of maternal mtDNA carried, from 4% (passage 1) until 60% (passage 12), although the cause could not be determined [36]. This phenomenon also occurred with long cultures of ESCs derived from embryos with spindle transfer.

The unique human gestation derived from a PNT was described by Zhang et al. 2016 [35]. The aim of this work was not to prevent a mitochondrial pathology, but to improve the fertility of a patient who had suffered early embryo deaths. Fetuses died prematurely due to cord prolapse and did not lead to a live birth. Zhang et al. reported a good rate of embryo development (Table 6) but the results were not directly comparable to those of other PNT publications on humans [20,36,37] since this study only informed about four-cell embryos (previous stage to blastocyst). Mitochondrial carry-over was undetectable, although this could be due to the employed measurement technique (not specified by the authors).

Undoubtedly, PNT has proved to be compatible with correct embryo development in mice and humans, although the levels of mitochondrial carry-over reported greatly differ among studies. As in the spindle transfer technique, the drift of carried mitochondrial DNA is a concern that must be investigated in detail. Furthermore, long-term studies to evaluate the transfer safety in successive generations will be required.

### 2.5. Polar Bodies Transfer (PB1T/PB2T)

Polar bodies appear as result of asymmetric cell divisions occurring during meiosis.

**Figure 6:**
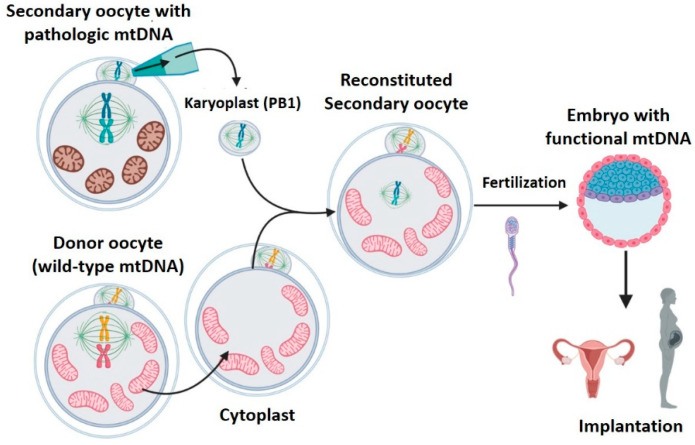
First polar body transfer (PB1T). PB1T consists of transferring the first polar body from a patient’s oocyte in metaphase II to a donor oocyte (also in metaphase II) whose meiosis spindle has been previously removed. Created with BioRender.com.

**Figure 7:**
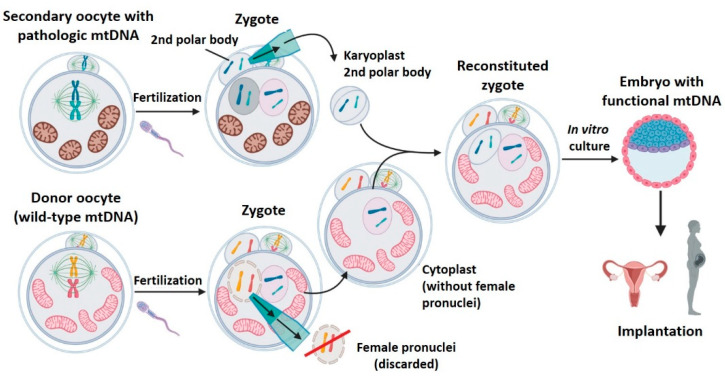
Second polar body transfer (PB2T). PB2T consists in transferring the PB2 from a patient’s zygote with a mtDNA mutation to a donor zygote whose female pronucleus has been previously removed. Created with BioRender.com.

- First polar body transfer (PB1T): at the end of meiosis I, a secondary oocyte (containing most of the primary oocyte cytoplasm) and the first polar body, which is extruded to the cell periphery, are obtained. The secondary oocyte and first polar body genomes are practically identical [31]. That is, formed by 23 chromosomes (n) with two chromatids each (2c). Unlike the metaphase spindle of the secondary oocyte, the first polar body spindle is clearly delimited by a surrounding plasmatic membrane, with a low amount of cytoplasm. The first polar body transfer (PB1T) consists of transferring the first polar body from a patient’s oocyte in metaphase II to a donor oocyte, also in metaphase II, without meiosis spindle (previously removed) [31,38,39,40] (Figure 6). The resulting oocyte will be fertilized and implanted. This technique is very similar to MST, but the meiotic spindle in the recipient enucleated oocyte is replaced by the first polar body.
- Second polar body transfer (PB2T): when the secondary oocyte is fertilized, meiosis II ends to form a female pronuclei whose chromosomes have one unique chromatid (from (n, 2c) to (n, c)). The second meiotic division also generates the second polar body (PB2) whose genetic material is similar to that of the zygote’s female pronuclei (n, c). As occurs with PB1, PB2 is well delimited in the zygote’s periphery and has little cytoplasm. PB2T consists in transferring the PB2 from a patient’s zygote to a donor zygote whose female pronuclei has been previously removed (since it contains donor nDNA) [31,38,40] (Figure 7). As in PNT, both PB1T and PB2T require zygote manipulation. First polar body transfer (PB1T) consists in transferring the first polar body (PB1) from the secondary maternal oocyte to a donor oocyte previously enucleated (without meiotic spindle). Thus, this technique is similar to mother spindle transfer. In contrast, second polar body transfer (PB2T) implies isolating the second polar body (PB2) of a maternal zygote (PB2) and substituting the female pronuclei of a donor zygote. PB2T is similar to PNT since both of them use zygotes.

PB1T and PB2T were proposed as MRT by Wang et al. 2014 [31]. The authors demonstrated that polar bodies contain the necessary genetic information to carry out the embryo development in mice. Furthermore, they compared the efficacy of PB1T and PB2T with that of MST and PNT, respectively. The PB1T efficacy of blastocyst development was up to 82.4%, and achieved a birth rate of 42.8%. PB2T yielded slightly lower efficacies in blastocyst development than PB1T and controls. The main advantage of these techniques is the low rate of carry-over, especially in PB1T, when compared with the other strategies. Moreover, mitochondrial carry-over remained low for two generations in mice (Table 7). Employing polar bodies implies lower intrinsic carry-over since they are already surrounded by a membrane and contain very little cytoplasm [31]. Another advantage proposed by the authors for PBTs is that the amount of genetic material available would increase. One maternal oocyte could provide the spindle and PB1, and one zygote could provide the pronuclei and PB2 [31]. This would reduce the number of biological mothers required to generate offspring, but this potential advantage was not evaluated in any studies.

PB1T and PB2T proved to be viable in human gametes too (Table 7) [38,39,40]. The potential of embryo development of PB1T was evaluated in all the studies selected, and the results obtained were acceptable. PB2T showed contradictory results in the different works: whereas Zhang et al. [38] were not able to generate blastocysts, Wu et al. [40] obtained similar data to those observed with PB1T. These variable results were probably due to a technical difficulty in PB2T: unlike in mice, it is very difficult to identify the female pronuclei in human zygotes. Only one work studied the mitochondrial carry-over in humans employing PBTs, and reported minimal levels in both embryos and ESCs (Table 7) [40]. In addition, this carry-over remained low and was stable for prolonged culture (20 passages) of ESCs [40]. This contrasts with the reversion to the carried mitochondrial haplotype commented on with spindle [32,33] and pronuclei transfer [36], suggesting that minimal levels of mitochondrial carry-over would prevent the appearance of this unpredictable phenomenon. One of the major objections to the use of polar bodies is that the nuclear genome and the polar bodies (discarded from meiotic divisions) could have epigenetic differences. However, two studies demonstrated that the genetic information of the oocyte–zygote and polar bodies is nearly the same, and their epigenetic environment is very similar too [31,39].

In summary, PBTs have proved to be compatible with embryonic development in animals and humans, with minimal levels of mitochondrial carry-over, the lowest among the MRTs. However, further long-term studies would be required to confirm that these techniques are able to generate healthy individuals without epigenetic anomalies, and to determine the real efficacy of PB2T, given the technical difficulties in human zygotes.

### 2.6. Benefits and Risks of Mitochondrial Procedure

#### 2.6.1. Comparison of Mitochondrial Replacement Techniques (MRTs)

We could only find three studies (in murine models) comparing different strategies of mitochondrial replacement, but no one has compared simultaneously the five techniques described [27,28,31]. Germinal vesicle transfer obtained poor results in embryonic development, discounting its use in a clinical setting in the near future. Regarding the carry-over, whereas Neupane et al. [28] obtained very low levels and did not find significant differences among the different strategies, Wang et al. [31] reported abnormally high rates with meiotic spindle transfer and pronuclei transfer. Thus, the results found in the literature show great discordance, and there is a lack of articles comparing all these techniques in the same animal model. In addition, no study has compared the results with those obtained in human oocytes and, thus, these might not be applicable. The preference of using a specific MRT seems to depend on the research team, usually based on their previous publications (MST for Mitalipov [19,30,33] and Egli [29,32] teams in the US, and PNT for the Herbert group in the UK [20,36]. Each of these groups specialized in a technique and they have not published comparative works testing the different MRT procedures within the same model and laboratory. These kinds of studies will be required in the future to determine which is the most efficacious technique. MST and PNT have proved to be compatible with normal embryo development, even in human gestations [34,35], and jointly they have accumulated the highest number of publications. Although PNT tends to generate slightly higher rates of mitochondrial carry-over [31,32,35,37], there is not enough evidence supporting the choice of one over the other [28]. In that case, the ethical objections about PNT, since it requires generating a zygote with a donor oocyte and discarding its genetic material, could unbalance the choice towards MST, as occurred in the case of the male child birth described in Zhang et al. 2017 [34] (although the authors had more experience in PNT). As described above, PBTs have minimal carry-over rates [31,40] and could potentially reduce the number of biological mother oocytes required to conceive when compared to MST and PNT [31]. Despite the advantages of these techniques, there have not been many publications employing them. In addition, only one work compared the PBTs with the rest of techniques and, for this reason, further studies will be required prior to their clinical application. Given the lack of clarifying results that permit identifying the optimal strategy, the main advantages and disadvantages of each transfer technique have been compiled in Table 8.

It must be underlined that the use of ESCs for experimental purposes offers many advantages but also disadvantages. Besides the ethical implications, it is a new strategy and further studies are required to evaluate their efficiency and safety, since they could be rejected if used in transplants, and their direct use from undifferentiated ESC could cause tumors. Furthermore, ESCs can serve as an ex vivo model, but their behavior is not exactly the same as normal embryos.

#### 2.6.2. Risks Associated to Mitochondrial Replacement Therapies

The scientific community has proposed three potential problems associated with MRTs that will be described in the following section:

1. Risks derived from micromanipulation
2. Mitochondrial carry-over
3. Mito-nuclear incompatibility

Risks Derived from Micromanipulation

These include those risks secondary to the oocyte handling within the laboratory:

(a) Sendai virus extract (HVJ-E): it is employed to fuse the karyoplast with the cytoplast. Although this extract is purified and only contains envelope proteins, it could contain traces of viral genome RNA with the potential to integrate in the embryo genome [19]. The possible repercussions of this potential scenario are unknown. The rhesus macaques that were born employing spindle transfer did not have the presence of viral genetic material within their genome [19]. However, there remains some reticence to using viral proteins as fusogens in a clinical setting [38]. In this sense, Zhang et al. [34] chose the electrofusion procedure for their spindle transfer intervention that led to the first healthy human male birth, aiming to avoid introducing foreign proteins [34]. More information about its safety will be required prior to its clinical translation.
(b) Cytoskeletal disruptors (cytochalasin B, nocodazole): during nuclear transfer procedures, the oocytes are incubated with cytoskeleton disruptors to facilitate their manipulation [19,20]. Although they are also used frequently in techniques of in vitro fertilization, their possible deleterious effects have not been evaluated properly [37]. Only Wu et al. [37,40] modified the transfer procedures to avoid the administration of disruptors, obtaining positive results.
(c) Nuclear genome damage: instrumental stress over oocyte genetic material could cause chromosomal damage and increase embryonic aneuploidies [38]. This could be more probable when transferring the meiotic spindle, because it is not protected by a membrane, however no information about this was found.

Mitochondrial Carry-Over

Mitochondrial carry-over is the mtDNA inexorably transferred from the biological mother oocyte jointly with the nuclear DNA to a donor oocyte. This causes cells of the future individual to have two different mtDNA: one from the biological mother and the other from the donor, this is known as heteroplasmy. This has been explained previously. The problem of carry-over in the 2016 works [32,33,36] generated a paradigm shift about MRTs: it was demonstrated that these techniques were not able to completely prevent the transmission of mitochondrial diseases (mtDNA), although they reduced their probability. It must be a priority in future research to look for methods to reduce this carry-over to its minimum (by transferring polar bodies, for example) and, especially, increase the knowledge about in vivo cycle of mtDNA, to be able to foresee the probability of an individual suffering from a mitochondrial disease. For this reason, it will be necessary to follow-up the individuals born with these techniques over long periods of time (both animals and humans).

Mito-Nuclear Incompatibility

As previously stated, in spite of the existence of mtDNA, many proteins that play an important role in mitochondrial functions are encoded in nDNA. Then, the correct function of mitochondria (electron transport chain, mtDNA replication, etc.) depends on the coordination among the proteins encoded by the nuclear and mitochondrial DNA [43,44,45]. With very few exceptions, eukaryotic organisms exclusively inherit their mitochondria from their mother [46]. Since it is a reproductive mechanism preserved in all the phylogenetic tree, it is probable that it has played a crucial role in evolution. For this reason, some authors hypothesize that mitochondrial and nuclear genomes have undergone a process of evolutionary co-adaptation [41,47,48], favoring those most advantageous mito-nuclear combinations (between nDNA and mtDNA). When a MRT is performed, this uniparental inheritance mechanism is broken: the nDNA is located in a completely new environment, with alien mitochondrial genes and with the risk of generating incompatibilities derived from these new nDNA–mtDNA associations. This hypothesis has been proved in cybrid (cytoplasmic hybrids, with nDNA and mtDNA from different origins) animal models, in which the mito-nuclear incompatibilities generated problems such as growth delay and low activity of enzymes involved in oxidative phosphorylation [41,45,48,49,50]. The research groups that work on MRTs allege that the presence of mito-nuclear incompatibilities in humans is improbable, mainly due to the exogamic nature of our species [51]. In fact, it has been suggested that MRTs are not different to what occurs daily in a society when individuals from different ethnicities have offspring [49,51]. Nevertheless, some studies have tried to compare nuclear and mitochondrial DNA sequences collected from human populations in order to detect possible mito-nuclear incompatibilities, with contradictory results. The presence of nuclear and mitochondrial DNA proceeding from different ancestral origin has been associated to an increased risk of preterm labor [44] and reduced amount of intracellular mtDNA (which has been related to different pathologies, such as Parkinson’s) [45]. In contrast, another research work did not find mito-nuclear combinations with deleterious effects in the general population [49]. We found only two systematic reviews/meta-analysis that evaluated the risk of mito-nuclear incompatibilities derived from the clinical use of MRTs, joining results from studies in invertebrate and vertebrate cybrids [48,50]. Again, the results were discordant. On the one hand, Eyre-Walker 2017 [50] estimated a negligible risk of mito-nuclear incompatibilities derived from MRTs in humans. On the other hand, Dobler et al. 2018 [48] concluded that incompatibility risks exist in, at least 1 out of 130 individuals born employing these techniques. The authors of both works have harshly criticized each other’s study, especially the methodology and statistical calculation, making it more difficult to draw conclusions. Regardless of mito-nuclear incompatibilities’ existence in humans, this problem could be avoided by selecting those donor oocytes whose mitochondrial haplogroup was identical to that of the biological mother (haplocompatibility). This would not only attenuate the risk of mito-nuclear incompatibilities, but it would also avoid the carried maternal mtDNA having a replicative advantage against the donor mtDNA. However, the search for haplocompatible oocytes is a logistical problem for the clinical application of MRTs, since donor ovules must be fresh and cannot be stocked cryogenized until their use (cytoplasts do not resist freezing [30,32,33]).

### 2.7. Ethical and Legal Aspects of Mitochondrial Replacement

Mitochondrial replacement techniques imply the manipulation of human gametes in a similar way to cloning techniques (somatic cell nuclear transfer). For this reason, the potential clinical application of these procedures has generated ethical, religious, and legal objections [9,23]. In the present work, we have tried to present the main concerns that have been the focus of ethical-legal discussion of MRT by answering four questions:

1. Do MRTs constitute a germinal gene therapy?
2. Do the individuals born by MRTs have three parents (2 mothers and 1 father)?
3. Are MRTs ethically justifiable?
4. What is the legal status of MRTs? What are their indications?

#### 2.7.1. Should MRTs Be Considered a Germinal Gene Therapy?

There is global scientific consensus against germline (gametes, zygotes) genetic modifications [52], and this is mainly due to the fact that the changes introduced within an individual are inheritable and will affect future generations (with unpredictable consequences) [53,54]. However, the term “germinal gene therapy” is usually used to define changes in nuclear genome, not in mitochondrial DNA [23,55]. The authors in favor of MRTs allege that nuclear transfers are “inheritable modifications” but not an intervention in the germline, since they do not affect the nuclear DNA (which contains the information that determines the characteristics of an individual) [9,54,55,56]. In addition, they argue that mtDNA constitutes a tiny part of the total cell genome (0.1%). To other authors, this is an excessive simplification of the mtDNA’s impact on phenotype and personal identity [23,53]. Mitochondrial DNA defines matrilineal genetics and it has a paramount importance in some ethnicities. In any case, MRTs are at the limit of being considered germline interventions: whether or not a male is born by an MRT, he will not provide his mitochondrion to the offspring, similarly to what occurs with somatic gene therapy. The reticence to use MRT because of its risk in affecting the germline could be solved by selecting only the male embryos [54] until data about the long-term safety of these techniques are available. In fact, the long-term follow-up could constitute a limiting regulatory system to prevent other germline interventions being progressively performed (slippery slope argument).

#### 2.7.2. Are They Really Children of 3 Parents?

The general press often uses the term “three parent baby” to refer to individuals born by mitochondrial replacement techniques [57]. It is a true denomination sensu stricto since the children have genetic material from three different persons. However, MRTs defenders have criticized the use of this expression for being sensationalist and influencing public opinion [53,57]. Three-parent baby is an unfortunate denomination, and does not fully reflect the reality: the paternity/maternity relation has a social nuance of care and protection that the baby would never have with the oocyte donor, although she contributed genetically [23,57]. In the same way that adoptions and ovum donor gestations are permitted, affirming that MRTs children have three parents seems unfair and senseless.

#### 2.7.3. Are MRTs Ethically Justifiable?

Today, there is no curative treatment for mitochondrial pathologies. The therapeutic approach is limited to treating the symptoms and giving support [58]. From the perspective of women carrying mutated mtDNA [59] and for those clinicians specialized in mitochondrial diseases [9,51,58], the application of MRTs to prevent the transmission of pathologic mtDNA is ethically justifiable.

No therapeutic intervention is free of risk, especially during the first clinical translations [56]. MRTs could alleviate the suffering of those families whose children have passed away because of mitochondrial fatal diseases. However, a benefit/risk balance must be the goal [25]: the safety of future generations must be a priority. For the UK Human Fertility and Embryology Authority [9,26] and the US Institute of Medicine [54], once MRTs have proved safe enough in preclinical models, the first human trials could start. Not all the authors publishing about bioethical topics approve the use of MRTs. For example, F. Baylis considers that having genetic links with children is a desire and not a need, so mtDNA mutation carriers could look for other maternity methods, such as the adoption [53]. However, the desire for having genetically related offspring is common and strong [52]. Another frequent argument against MRTs is that of the “slippery slope” [23,52], which consists in: whether MRTs are approved and legalized, the society is a step closer to gene edition and “embryos on demand” [53]. This could be mitigated by creating legal barriers that limit the cases in which MRTs are permitted, as occurs in the UK [25]. Like in any other therapeutic intervention, it is crucial that women submitted to MRT in the future are well informed about all the reproductive alternatives available to be able to decide what they prefer.

#### 2.7.4. Which Is the Legal Status of MRTs? What Are Their Indications?

In 2015, the UK became the first and only country in the world to legalize the use of MRTs, after years of public consultations and Parliament deliberations [9,60,61]. Only spindle and pronuclei transfers are permitted, after authorization, in specific cases of demonstrated risk that offspring could inherit a serious mitochondrial pathology [60]. Furthermore, the performing clinics must have a long-term follow-up plan for the children born by MRT [9,61]. Gender selection is not considered, since reducing the number of available embryos for implantation would diminish the efficacy of the procedure [9]. No birth has yet occurred, although two licenses have already been granted for PNT in the Newcastle group that has extensively worked on this technique [20,36]. The public and political debate generated in the UK is a great model to follow. Except for the US, where any inheritable modification in germinal cells is explicitly forbidden [61], there is a global lack of specific legislation about MRTs, due to its borderline position. Surprisingly, the first human birth by a MRT did not take place within the UK but in the US in April 2016 [16]. This event agitated the scientific community: Dr. Zhang [34] took advantage of the lack of legislation about MRTs in Mexico to implant there an embryo that was created in the US by spindle transfer [16]. In other words, the procedure occurred in a trans-national manner to avoid the US legal restrictions. This is the antithesis of how scientific progress should be promoted and, thus, it was seriously criticized [26,60]. Recently, private fertility clinics have taken advantage of this status of legal void of MRTs in Europe, and two clinical trials have begun, one in Ukraine (led by Dr Zhang, [62]) and another one in Greece [63]. Both trials have reported child births during 2019 and more are expected in the near future. The lack of a therapeutic aim is the most alarming point, since these two trials are being employed to improve the fertility in elderly women with failure in several in vitro fertilization cycles [62,63]. However, there is not enough scientific evidence supporting the role of mitochondrion in infertility [23] of uncertain etiology. For this reason, this clinical application should not be adopted until more information about its safety in families carrying mutated mtDNA is compiled [60]. In this sense, the European Society of Human Reproduction and Embryology (ESHRE) has made a call for caution when applying these techniques in infertility cases.

## 3. Conclusions

As a general conclusion, it has been demonstrated that mitochondrial replacement techniques are a viable alternative for women carrying mtDNA mutations who desire to have disease-free genetically related offspring. In addition, the information compiled in the present review permits us to conclude specifically that:

1. Germinal vesicle transfer is not efficient in human oocytes.
2. Spindle transfer has accumulated a wide experience, including human gestations.
3. Pronucleus transfer is an efficient technique, extensively described in the literature, but presents ethical concerns as it requires zygote destruction.
4. First polar body transfer is a promising strategy with excellent preclinical results, but further studies will be required prior to its application.
5. Second polar body transfer presents technical difficulties in human zygotes.
6. MRTs reduce the risk of vertical transmission of mitochondrial diseases, but they do not completely prevent it. For this reason, it is recommended:
  - To minimize the mitochondrial drag by choosing those procedures with the lowest intrinsic carry-over (polar bodies).
  - To use oocytes from donors with a compatible haplogroup.
  - Selecting male embryos.
  - Long-term follow-up of animals and humans born by MRTs.
7. It is mandatory that governments legislate about the use of MRTs, especially to define those clinical applications in which the risks surpass the potential benefits.

## Figures and Tables

**Table 1:** Mitochondrial replacement technique comparison.

Procedure/ Characteristics	GVT	MST	PNT	PB1T	PB2T
Origin	Primary oocyte	Secondary oocyte	Zygote	Secondary oocyte	Zygote
Ploidy	Diploid (2n)	Haploid (n)	Diploid (2 × n, mas. and fem.)	Haploid (n)	Haploid (n)
Chromatids	4C	2C	2 × C (masc. and fem.)	2C	C
Surrounding membrane	+	-	+	+	+

‘n’ represents the number of chromosome sets. ‘C’ is the amount of chromatids in a chromosome pair. GVT: germinal vesicle transfer. MST: meiotic spindle transfer. PB1T/PB2T: transfer of first/second polar body. PNT: pronucleus transfer.

**Table 2:** GVT results in murine model.

Ref.	Transfer Method	Blastocyst Development	Births Rate	Carry-Over	Notes
Cheng et al., 2009 [31]	Electrofusion (EF)	Not indicated	7.7%	No tested	Lower post-natal growth
Neupane et al., 2014 [36]	HVJ-E	Inter-strain: 0% (0/14) Intra-strain: 0% (0/8)	N/D	Reconstructed oocytes: 0% (0/20)	MST and PNT were also compared

HVJ-E: Sendai virus extract. N/D: not determined.

**Table 3:** Results of MST in animal models.

Ref.	Transfer Method	Blastocyst Development	Births Rate	Carry-Over	Notes
Tachibana et al., 2009 [19]	HVJ-E EF	HVJ-E: 61% (45/74) similar to control EF: 9% (1/11) lower than control	27% (4/15) 17% Similar to control	ESCs and offspring: undetectable (<3%)	First animals born by MST Lower development to blastocyst due to premature oocyte activation
Cheng et al., 2009 [27]	EF	Not indicated	Inter-strain: 26% Intra-strain: 34% Similar to control	Not tested	GVT and PNT also performed
Neupane et al., 2014 [28]	HVJ-E	PTGN: 82.6% (19/23) similar to control ICSI: 0% (0/12) similar to control	Not tested	0.29% ± 0.63% Undetectable in 17/24	Carry-over of MST, GVT, and PNT were compared
Wang et al., 2014 [31]	HVJ-E	85.7% (18/21)	44.4% (8/18)	F1 tail: 5.5% ± 1.4% F2 fingers: 7.1% ± 6.8%	First publication to propose polar bodies transfer

All the works, except the Tachibana group (that was carried out in rhesus macaques), were performed in murine model. ESCs: embryonic stem cells. EF: electrofusion. F1/F2: mice of first and second generation. HVJ-E: extract of de Sendai virus. ICSI: intracytoplasmatic spermatozoid injection. PTGN: partenogenetically activated oocytes (without fertilization).

**Table 4:** Results of MST in human.

Ref.	Fertil. Proc.	Transfer Method	Blastocyst Development	Births Rate	Carry-Over	Notes
Tachibana et al., 2013 [30]	ICSI	HVJ-E	43% (19/44) lower than control	Not tested	Embryos: 0.5% ± 0.4% ESCs: 0.6% ± 0.9%	First MST in human oocytesAbnormal fertilization in 52% zygotes
Paull et al., 2013 [29]	PTGN	HVJ-E EF	38.9 (7/18) 33% similar to control	Not tested	Embryos: 0.31% ± 0.27% ESCs: <0.5% * * except 1 line (P4-P14) 2.79%	Prevention of spindle activation by low-temperature electrofusion
Yamada et al., 2016 [32]	PTGN	Not indicated	32% (cryogenized karyoplast + fresh cytoplast)	Not tested	Embryos: 0.2% ESCs: 0% in 7/8 cell lines (P6-P30) In 1/8 cell lines P0-1%; P36 = 53%; P59 = 1%	Complete reversion to biological mother mitochondrial haplotype in ESCs derived from SCNT
Kang et al., 2016 [33]	ICSI	HVJ-E	Healthy: 62.5% (20/32) Mutation carriers: 50% (6/12)	Not tested	Embryos: <1% ESCs: 15/18 <1% 3/18 100%	Ovum from pathological mtDNA carriers Improved abnormal fertilization
Zhang et al., 2017 [34]	ICSI	EF	80% (4/5)	100% (1/1)	Blastocyst: 5.10% ± 1.11% Urine: 2.36% Mouth: 5.59% Foreskin: 9.23%	First human birth by mitochondrial replacement technique

ESCs: embryonic stem cells. EF: electrofusion. F1/F2: mice of first and second generation. HVJ-E: extract of de Sendai virus. ICSI: intracytoplasmatic spermatozoid injection. PTGN: partenogenetically activated oocytes (without fertilization). SCNT: somatic cells nuclear transfer. P: passage.

**Table 5:** Results of PNT in mice.

Ref.	Transfer Method	Blastocyst Development	Births Rate	Carry-Over	Notes
McGrath et al., 1983 [42]	EF	96% (64/67) Similar to controls	15% (10/64) Similar to controls	Not tested	First publication about PNT
Sato et al., 2005 [43]	EF	Not indicated	28% (11/39) Similar to controls	F1 tail: 11% (6–21%) After 300 days: 23% (5–44%)	Murine model with mitochondrial pathology. Offspring free of disease
Cheng et al., 2009 [27]	EF	Not indicated	31–62%	Not tested	Animals born were healthy and comparable to controls
Neupane et al., 2014 [28]	HVJ-E	87.5% (14/16) Similar to non-manipulated controls	Not tested	Embryos: 0.29% ± 0.75% Undetectable in 21/25	Minimum levels of carry-over. Equivalent to those of GVT and MST
Wang et al., 2014 [31]	HVJ-E	81.3% (13/16) Similar to controls	53% (7/13) Similar to controls	F1 tail: 23.7% ± 11.1% F2 fingers: 22.1% ± 18.7%	High rates of carry-over, probably due to manipulation problems

ESCs: embryonic stem cells. EF: electrofusion. F1/F2: mice of first and second generation. HVJ-E: extract of de Sendai virus. ICSI: intracytoplasmatic spermatozoid injection.

**Table 6:** Results of PNT in humans.

Ref.	Transfer Method	Blastocyst Development	Births Rate	Carry-Over	Notes
Craven et al., 2010 [20]	HVJ-E	8.3% lower than control	Not tested	Embryos: 8.1% ± 7.6% Embryos (careful manipulation): 1.68% ± 1.81%	Employed abnormally fertilized embryos
Hyslop et al., 2016 [36]	HVJ-E	Heterologous PNT: approx. 39% Lower than non-manipulated controls (63%) and autologous PNT (60%)	Not tested	Embryos (modified technique): <5% (<2% in 79%; 2–5% in 21%) ESCs: <2% in 4/5 0–60% in 1/5	Early PNT and modified manipulation medium. Higher quality blastocysts
Zhang et al., 2016 [35]	EF	4-cell embryo: 71.4% (5/7)	Implantation: 60% (3/5) Births: 0% deaths by umbilical cord collapse	Fetal red cells: undetectable (undetermined sensitivity)	Reported a 2003 case
Wu et al., 2017 [37]	HVJ-E	25.64% (13/64) similar to non-manipulated controls	Not tested	Embryos: 1 ± 1.45% ESCs: 0.26 ± 0.17% (P5-P20)	Employed pre-pronuclei without cytoskeleton disruption

ESCs: embryonic stem cells. EF: electrofusion. P: cell culture passage. HVJ-E: extract of de Sendai virus Same group works have been colored with same color.

**Table 7:** Results of PBT in animals and humans.

Ref.	Species	Transfer Method		Blastocyst Develop	Births Rate	Carry-Over	Notes
Wang et al., 2014 [31]	Mice (NZW and BDF1)	HVJ-E	PB1T	82.4% (14/17) similar to MST	42.8% (6/14) similar to MST	F1 tail: 0% (0/6) F2 fingers: 0% (0/16) lower to MST	Compared PB1T with MST and PB2T with PNT
			PB2T	40% (6/15) lower than PNT	40% (6/15) similar to PNT	F1tail: 1.7 ± 2.8% F2 fingers: 2.9 ± 4.3% lower than PNT	
Ma et al., 2017 [39]	Humans	HVJ-E	PB1T	32.5% (8/25) lower than control	Not tested	Not tested	Same group of refs: [19,30,33]
Zhang et al., 2017 [38]	Humans	HVJ-E	PB1T	24.1% (7/29) similar to control	Not tested	Not tested	Data of fresh and cryogenized karyoplast transfers were added up
			PB2T	0% (0/17) lower than control	Not tested		
Wu et al., 2017 [40]	Humans	HVJ-E	PB1T	25.3% (19/75) similar to control and PB2T	Not tested	Embryos: 0.26% (0.07–0.5%) ESCs: 0.15% (P5-P20)	Good results due to performing the transfer right after the fertilization (pre-pronucleus) Same group of ref. [37]
			PB2T	27.5% (14/51) similar to control and PB1T	Not tested	Embryos: 0.37% (0.06–0.7%) ESCs: 0.22% (P5-P20)	

HVJ-E: Sendai virus extract. PB1T: first polar body transfer. PB2T: second polar body transfer. MST: meiotic spindle transfer. PNT: pronucleus transfer. ESCs: embryonic stem cells. F1: first generation. F2: second generation.

**Table 8:** Main advantages and disadvantages of MRTs.

MRT	Advantages	Disadvantages
**GVT**	- Embryo generation not required - Germinal vesicle is large and easy to manipulate	- Very low efficacy (requires in vitro maturation) - Deficient growth in murine model
**MST**	- Embryo generation not required - Spindle has few surrounding mitochondrion (low carry-over) - Large number of articles with good results	- Meiotic spindle is hard to visualize (not surrounded by membrane) - Possibility of not transferring a chromosome disperse within the cytoplasm (frequent in elderly women’s oocytes) - Spindle is sensitive to micromanipulation (maturation premature activation)
**PNT**	- Pro-nuclei resist micromanipulation - Pro-nuclei are easily visible under the microscope (surrounded by membrane) - Large number of publications	- Generation and destruction of an embryo (donor zygote) - Carry-over rate depends on the operator (cytoplasm surrounding the pro-nucleus is rich in mitochondrion)
**PB1T**	- Embryo generation not required - Minimum carry-over - Easy manipulation of PB1 (isolated in a membrane) - Possible to combine with MST (saving maternal ovules)	- Possible epigenetic differences between PB1 and oocyte genomes ** - Lack of publications
**PB2T**	- Minimum carry-over - PB2 is easy to manipulate (isolated in a membrane) - Possible to combine with PNT (saving maternal ovules)	- Generation and destruction of an embryo (donor zygote) - Possible epigenetic differences between PB2 and oocyte genomes ** - Lack of publications - Poor efficacy: difficult to distinguish female pro-nucleus in human zygotes

** This disadvantage has been refuted by recent works [31,39], although further data will be required. MRT: mitochondrial replacement techniques. GVT: germinal vesicle transfer. MST: meiotic spindle transfer. PNT: pro-nucleus transfer. PB1T: first polar body transfer. PB2T: second polar body transfer.

## Data Availability

Not applicable.
